# Multi-marker approach using procalcitonin, presepsin, galectin-3, and soluble suppression of tumorigenicity 2 for the prediction of mortality in sepsis

**DOI:** 10.1186/s13613-017-0252-y

**Published:** 2017-03-07

**Authors:** Hanah Kim, Mina Hur, Hee-Won Moon, Yeo-Min Yun, Salvatore Di Somma, on behalf of GREAT Network

**Affiliations:** 1 Department of Laboratory Medicine, Konkuk University Medical Center, Konkuk University School of Medicine, 120-1, Neungdong-ro, Hwayang-dong, Gwangjin-gu, Seoul, 05030 Korea; 2 Departments of Medical-Surgery Sciences and Translational Medicine, School of Medicine and Psychology, Sant’ Andrea Hospital, ‘Sapienza’ University, Rome, Italy

**Keywords:** Sepsis, Prognosis, Procalcitonin, Presepsin, Galectin-3, sST2

## Abstract

**Background:**

Biomarker could be objective and reliable tools to predict mortality in sepsis. We explored the prognostic utilities of emerging biomarkers in septic patients and questioned whether adding biomarkers to the clinical variables would improve the prediction of mortality in sepsis.

**Methods:**

This retrospective study included 157 septic patients (112 patients with sepsis; 45 patients with septic shock). Procalcitonin (PCT), presepsin, galectin-3, and soluble suppression of tumorigenicity 2 (sST2) concentrations were analyzed in relation to the 30-day all-cause mortality. Their value added on top of Sequential (Sepsis-related) Organ Failure Assessment (SOFA) score, high-sensitivity C-reactive protein, and white blood cells was also analyzed.

**Results:**

PCT could not predict 30-day mortality. Univariate hazard ratio [HR with 95% confidence interval (CI)] of the other dichotomized variables was: 1.33 (0.55–3.194) for presepsin; 7.87 (2.29–26.96) for galectin-3; 1.55 (0.71–3.38) for sST2; and 2.18 (1.01–4.75) for SOFA score. The risk of 30-day mortality increased stepwise as the number of biomarkers above optimal cutoff values increased, and the highest risk was observed when all four biomarkers and SOFA score increased (HR = 14.5). Multi-marker approach predicted 30-day mortality better than SOFA score [area under the curves (95% CI), 0.769 (0.695–0.833) vs. 0.615 (0.535–0.692)]. In reclassification analyses, adding biomarkers to clinical variables improved the prediction of mortality.

**Conclusion:**

This study demonstrated a possible prognostic utility of PCT, presepsin, galectin-3, and sST2 in sepsis. Multi-marker approach could be beneficial for an optimized management of patients with sepsis.

## Background

Sepsis is a life-threatening organ dysfunction, identified as an acute change in total Sequential (Sepsis-related) Organ Failure Assessment (SOFA) score equal to or more than two points, caused by a dysregulated host response to infection, and septic shock is a subset of sepsis with profound circulatory, cellular, and metabolic abnormalities associated with increased mortality [1]. Sepsis is the primary cause of death from infection, especially if not diagnosed and treated promptly; therefore, urgent attention is mandatory. The Third International Consensus Definitions for Sepsis and Septic Shock (Sepsis-3) includes recommendations for laboratory testing to determine sequential organ dysfunction such as measuring white blood cells (WBCs) and differential, platelet counts, bilirubin, and serum creatinine (sCr) to determine progression of organ dysfunction for sepsis, and lactate concentrations for septic shock [1, 2].

Procalcitonin (PCT) has been known as a helpful biomarker for early diagnosis of sepsis, and the efficacy and safety of PCT-guided antibiotic treatment in critically ill patients in intensive care units (ICUs) have been proved [3]. In early 2016, the US Food and Drug Administration (FDA) expanded the clinical indications of PCT: the change in PCT concentrations over time as an aid in assessing the cumulative 28-day risk of all-cause mortality in conjunction with other laboratory findings and clinical assessments for patients diagnosed with septic shock in the ICU or when obtained in the emergency department or other medical wards prior to ICU admission [4, 5]. CD14 is a glycoprotein expressed on the surface membrane of monocytes/macrophages and serves as a receptor for lipopolysaccharides (LPSs) and LPS-binding proteins (LBPs). The complex of LPS-LBP-CD14 is released into circulation by shedding from the cell membrane, which is called soluble CD 14 (sCD14). Plasma protease generates cleaved sCD14, generating a truncated form of 64 amino acid residues named sCD14 subtype or presepsin [6, 7]. Presepsin revealed diagnostic and prognostic capacities to differentiate sepsis severity and to predict mortality in septic patients [8, 9]. Galectin-3 and soluble suppression of tumorigenicity 2 (sST2) have emerged as biomarkers in heart failure (HF) for additive risk stratification of patients with acute and/or chronic HF [10–12]. In addition to their association with HF, they can also increase in diverse non-cardiac conditions such as infectious diseases or chronic kidney diseases [13–15].

Given the profound circulatory, cellular, and metabolic abnormalities in sepsis with multiple organ dysfunctions, several biomarkers, if integrated together, may present more objective and reliable guide for the prognosis prediction in critically ill patients with sepsis. In the present study, we wanted to explore the prognostic utilities of multi-marker approach using PCT, presepsin, galectin-3, and sST2 in septic patients. We hypothesized that multiple biomarkers, in combination or alone, would predict mortality in septic patients. In particular, we questioned whether adding biomarkers to the clinical variables, such as SOFA score, high-sensitivity C-reactive protein (CRP), and WBC would improve the prediction of mortality in sepsis.

## Methods

### Study population

**Fig. 1:**
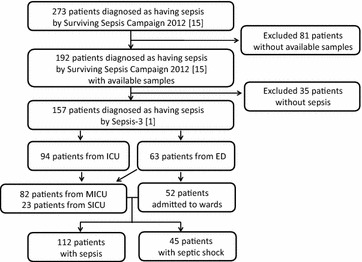
A flowchart for patient recruitment. Abbreviations: *ICU* intensive care unit, *ED* emergency department, *MICU* medical ICU, *SICU* surgical ICU

From December 2014 to June 2015, a total of 273 consecutive patients were diagnosed as having sepsis according to the Surviving Sepsis Campaign 2012 in the Konkuk University Medical Center, Seoul, Korea [16, 17]. Because we wanted to measure the biomarkers in leftover samples, 81 patients without available samples were excluded, and 192 patients with available samples were recruited. Because the definition of sepsis and septic shock was revised in early 2016, the 192 patients were recategorized according to the new Sepsis-3 definition [1]; 112 patients (58.3%) were diagnosed as having sepsis, 45 patients (23.4%) as having septic shock; and 35 patients (18.2%), who could not be included in sepsis category according to the new definition were excluded from this study (Fig. 1). For the remaining 157 patients, their medical records were reviewed retrospectively for the clinical and demographic data, including their comorbidities and treatment. They received the standard-of-care treatment according to the guidelines [18, 19]. The characteristics of the study population are summarized in Table 1.

**Table 1:** Characteristics of the study population

Variable	All patients (*N* = 157)
Sepsis criteria	157 (100.0)
Sepsis, *N* (%)	112 (71.3)
Septic shock, *N* (%)	45 (28.7)
Patients enrollment	
Intensive care unit, *N* (%)	94 (59.9)
Emergency room, *N* (%)	63 (40.1)
Age (years), median [IQR]	70 [57.7–77.0]
Males, *N* (%)	95 (60.5)
Hospital stay (days), median [IQR]	16 [8–40]
In-hospital mortality, *N* (%)	40 (25.5)
30-day mortality, *N* (%)	34 (21.7)
Comorbidities	
Hemato-oncologic, *N* (%)	31 (19.6)
Pulmonary, *N* (%)	29 (18.6)
Cerebrovascular, *N* (%)	28 (17.5)
Renal and genitourinary, *N* (%)	19 (12.4)
Gastrointestinal, *N* (%)	18 (11.3)
Cardiovascular, *N* (%)	16 (10.3)
Others, *N* (%)	16 (10.3)
Type of infections/proportion of infection episodes with isolated pathogens*	
Bacteremia, *N* (%)/%	90 (57.3)/100%
Respiratory infection, *N* (%)/%	102 (65.0)/88.2%
Urinary infection, *N* (%)/%	55 (35.0)/100%
Gastrointestinal infection, *N* (%)/%	26 (16.6)/46.2%
Others, *N* (%)/%	4 (2.5)/100%
eGFR by MDRD Study equation (mL/min/1.73 m^2^), median [IQR]	44.45 [20.83–81.33]
SOFA score range	2–11
	2 (45, 28.7%); 3 (32, 20.4%); 4 (26, 16.6%); 5 (14, 8.9%); 6 (13, 8.3%); 7 (12, 7.6%); 8 (6, 3.8%); 9 (3, 1.9%); 10 (3, 1.9%); 11 (3, 1.9%)
CRP (mg/dL), median [IQR]	12.54 [7.22–22.0]
WBC (× 10^9^/L), median [IQR]	12.47 [8.18–17.10]
PCT (ng/mL), median [IQR]	6.19 [2.25–21.99]
Presepsin (pg/mL), median [IQR]	2714.0 [1479.3–4129.7]
Galectin-3 (ng/mL), median [IQR]	30.8 [17.9–58.5]
sST2 (ng/mL), median [IQR]	214.5 [133.6–238.8]

* Multiple infections were observed in 112 patients (71.3%), and 20 patients (12.7%) had radiographically proven infection without pathogen isolation. The number of type of infections and proportion of infection episode with isolated pathogen is based on each infection episode

*IQR* interquartile range, *eGFR* estimated glomerular filtration rate, *MDRD* modification of diet in renal disease, *SOFA* sequential organ failure assessment, *PCT* procalcitonin, *sST2* soluble suppression of tumorigenicity 2

The protocol of this registry study was approved by the Institution Review Board (KUH1200051) of Konkuk University Medical Center, before collecting the first sample from the first patient. It was left open in the study protocol which biomarkers would be tested. This registry study required neither study-specific blood sampling nor other interventions. In all septic patients, PCT concentration was measured as a routine practice together with CRP, WBC, and sCr for estimated glomerular filtration rate (eGFR) at the day when patients were diagnosed as having sepsis; at the same day, SOFA score was assessed, and residual blood samples were collected for the measurement of the other biomarkers (presepsin, galectin-3, and sST2). Attending physicians (in ICU or ED) made the clinical diagnosis of sepsis according to the Surviving Sepsis Campaign 2012 and obtained blood samples for the routine measurements of PCT, CRP, and WBC; then, they informed the laboratory to store residual samples (both EDTA plasma and serum samples) from these blood collections. The samples were divided into small aliquots to avoid repeated freezing and thawing, and then stored at −70 °C until use. Frozen samples were thawed at room temperature and gently mixed up just before the measurement of biomarkers. Therefore, written informed consent from the patients was exempted.

### Assays

Serum PCT concentrations were determined as routine practice using the Elecsys BRAHMS PCT electrochemiluminescence assay (BRAHMS, Henningsdorf, Germany) on the Roche Cobas e-System (Roche Diagnostics, Basel, Switzerland). The other biomarkers were claimed to be stable at −70 °C up to 18 months by the manufacturer and was measured in August 2015 in one batch according to the manufacturer’s recommendations.

Plasma presepsin concentrations were measured using an automated chemiluminescent enzyme immunoanalyzer, PATHFAST system (LSI Medience Co., Tokyo, Japan). Presepsin in the sample binds to the anti-presepsin antibodies to assemble an immunocomplex with the ALP-labeled antibodies and the mouse monoclonal antibody-coated magnetic particles. After 10-min incubation with a chemiluminescent substrate, the luminescence generated by the enzyme reaction, photomultiplier detected and calculated the concentration of presepsin [6]. Plasma galectin-3 concentrations were measured using the VIDAS automated enzyme-linked fluorescent assay (bioMérieux, Marcy-l’Etoile, France). Serum sST2 concentrations were measured using the Presage ST2 Assay (Critical Diagnostics, San Diego, CA, USA). It is an enzyme-linked immunosorbent assay with mouse monoclonal anti-human sST2 antibodies coated 96-well microtiter plate [20]. The manufacturer-claimed measurable range of PCT, presepsin, galectin-3, and sST2 assays was 0.02–100 ng/mL, 20–20,000 pg/mL, 20–20,000, and 3.1–250 ng/mL, respectively. Coefficient of variation (CV) (%) of each assay was determined in our laboratory according to the CLSI document EP15-A2 [21]. The CVs were tested at two levels by running three replicates over five days; the CV of PCT, presepsin, galectin-3, and sST2 assays were <2.47%, <5.0%, <4.9%, and <3.0%, respectively.

The sCr was measured by the kinetic Jaffe method using Roche CREA (Roche Diagnostic, Mannheim, Germany) traceable to isotope dilution mass spectrometry (IDMS) on an automated chemistry analyzer TBA-200 FR (Toshiba Co., Tokyo, Japan). Dynamic measuring range was 0.2–25 mg/dL, and the mean within-laboratory precision of the sCr assay was 1.35% during the study period. eGFR was calculated by using the IDMS-traceable four-variable modification of diet in renal disease study equation [22]; GFR = 175 × sCr^−1.154^ × Age^−0.203^ × 0.742 [if female]. The high-sensitivity CRP was measured by CRP-Latex (II) X2 (Denka Seiken Co., Tokyo, Japan) by latex agglutination method on TBA-200 FR. Its measurement range was 0.01–35 mg/dL, and the mean within-laboratory precision was 2.0% during the study period. WBC was measured by an automated hematology analyzer XN modular system (Sysmex, Kobe, Japan). Its measurement range was 0.00–239.05 × 10^9^/L, and the mean within-laboratory precision was 0.85% during the study period.

### Statistical analysis

Data were expressed as median and interquartile range (IQR) or number and percentage. Groups were compared using Mann–Whitney *U* test. Receivers operating characteristic (ROC) curves of each biomarker and SOFA score were compared to derive optimal cutoff values for the prediction of 30-day all-cause mortality. Optimal cutoff values meant where the sum of false positive and false negative results were lowest. Areas under the curves (AUC) were reported with their 95% confidence interval (CI). Each biomarker and SOFA score were dichotomized (above and below cutoffs) according to the respective optimal cutoff values for 30-day all-cause mortality. Cox proportional hazard regression was used to analyze the effect of biomarkers and SOFA score on 30-day all-cause mortality; univariate hazard ratio (HR, with 95% CI) of the dichotomized variables was obtained. All 157 patients were divided into six groups (from 0 to 5) based on the frequency of above cutoff values, and each group was compared according to the 30-day mortality using Kaplan–Meier survival curves and HR (with 95% CI). With dichotomized variables using respective optimal cutoff values for 30-day mortality, ROC curves of SOFA score, combined biomarkers, and combination of SOFA score and biomarkers were generated again, and their AUC were compared for the prognostic utility of multi-marker approach. Reclassification analyses using net reclassification improvement (NRI) and integrated discrimination improvement (IDI) were used to assess the added value of multi-marker approach on top of SOFA score, CRP, and WBC; NRI and IDI values were analyzed with their 95% CI. For the statistical analyses, MedCalc Software (version 15.8, MedCalc Software, Mariakerke, Belgium) and R version 3.3.1 (The R Foundation for Statistical Computing, Vienna, Austria) were used. The *P* values were not adjusted for multiple comparisons and, therefore, were only descriptive.

## Results

**Fig. 2:**
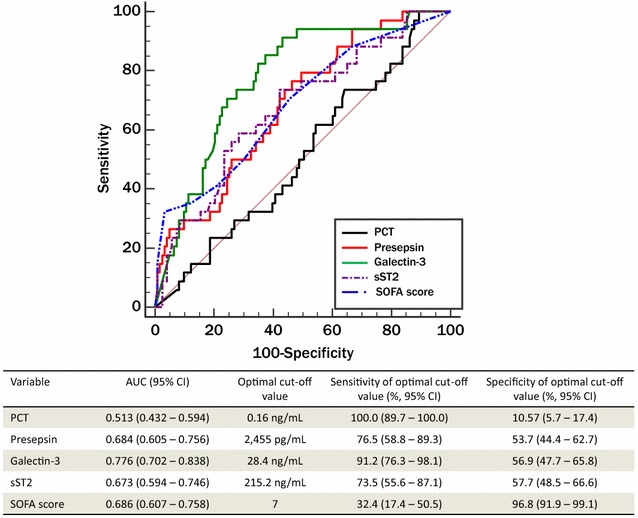
Comparison of the receiver operating characteristics curves to predict 30-day mortality in sepsis. For each biomarker and SOFA score, optimal cutoff values to predict 30-day mortality were obtained. Abbreviations: see Table 1

The concentrations of PCT, presepsin, galectin-3, and sST2 are presented in Table 1. Presepsin, sST2, and SOFA score were comparable for the prediction of 30-day all-cause mortality, and galectin-3 was superior to them with fair performance. PCT could not predict 30-day mortality. The optimal cutoff values for 30-day mortality were as follows: PCT, 0.16 ng/mL; presepsin, 2,455 pg/mL; galectin-3, 28.4 ng/mL; sST2, 215.2 ng/mL; and SOFA score, 7 (Fig. 2).

When the biomarkers and SOFA score were compared between the survivors and non-survivors, except for PCT concentration, the others were higher in the non-survivors than in the survivors (all *P* < 0.002), and univariate HR of the biomarkers of interest and SOFA score are given in Table 2.

**Table 2:** Comparison of PCT, presepsin, galectin-3, sST2, and SOFA score according to the 30-day mortality

	Total (*N* = 157)			30-day mortality	
	Survivor (*N* = 123)	Non-survivor (*N* = 34)	P*	HR (95% CI)^a^	*P*
Procalcitonin (ng/mL)	6.19 (2.24–22.39)	6.61 (2.22–20.78)	NS	–	–
Presepsin (pg/mL)	2,310.0 (1375.8–3920.2)	3,549.0 (2493.7–8242.7)	0.0011	1.33 (0.55–3.19)	NS
Galectin-3 (ng/mL)	24.5 (16.7–47.5)	58.6 (37.0–82.2)	<0.0001	7.87 (2.29–26.96)	0.0011
sST2 (ng/mL)	209.5 (116.9–236.9)	237.3 (208.8–253.3)	0.0020	1.55 (0.71–3.38)	NS
SOFA score	3 (2–5)	5 (3–8)	0.0007	2.18 (1.01–4.75)	0.0496

Data are expressed as median (interquartile range)

* Mann–Whitney *U* test

^a^Cox proportional hazard regression using dichotomized variables according to the respective optimal cutoff values for 30-day all-cause mortality derived from receiver operating characteristics curve analysis. HR was not analyzed for procalcitonin that showed no difference between survivors and non-survivors

See Table 1; *HR* hazard ratio, *NS* not significant

**Fig. 3:**
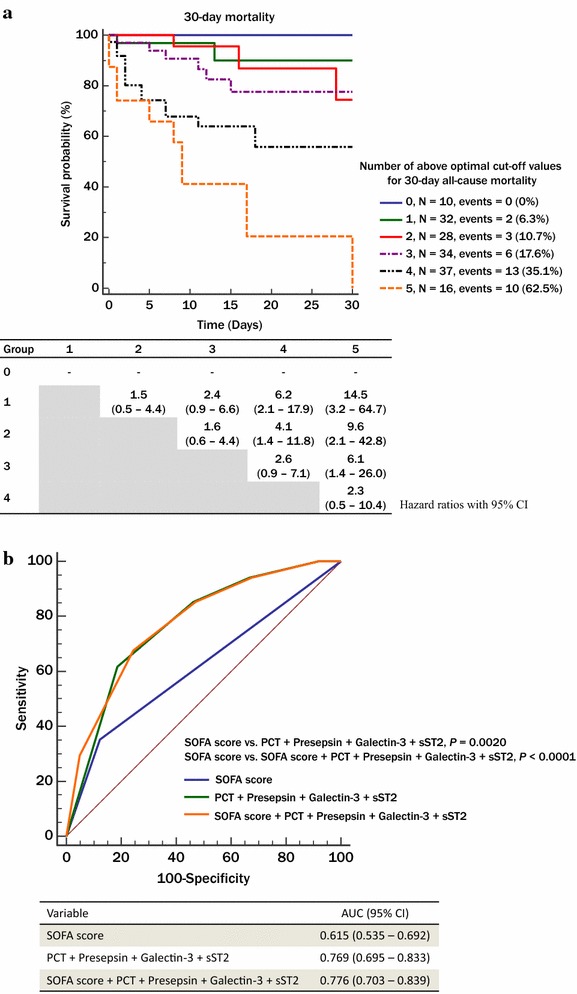
Multimarker approach to predict 30-day mortality in sepsis. **a** Multi-marker approach using above optimal cutoff values of PCT, presepsin, galectin-3, sST2, and SOFA score for the prediction of 30-day all-cause mortality. **b** Multi-marker approach using multivariate ROC curve analysis for the prediction of 30-day all-cause mortality. Abbreviations: *PCT* procalcitonin; sST2, soluble suppression of tumorigenicity 2, *ROC* receiver operating characteristics, *SOFA* sequential organ failure assessment, *IDI* integrated discrimination improvement, *NRI* net reclassification improvement, *CRP* C-reactive protein, *WBC* white blood cells, *CI* confidence interval

Multi-marker approach using above cutoff values of each biomarker and SOFA score showed differences for the prediction of 30-day morality. Mortality rate in each group showed a stepwise increase: 0% in group 0; 6.3% in group 1; 10.7% in group 2; 17.6% in group 3; 35.1% in group 4; and 62.5% in group 5. Group 5 showed higher HR compared with groups 1, 2, and 3; 14.5 (95% CI 3.2–64.7), 9.6 (95% CI 2.1–42.8), and 6.1 (95% CI 1.4–26.0), respectively (Fig. 3a). In ROC curve analysis, multi-marker approach predicted the 30-day mortality better than SOFA score [AUC = 0.769 (95% CI 0.695–0.833) vs. AUC = 0.615 (95% CI 0.535–0.692)]. Addition of SOFA score on multi-markers showed similar findings (Fig. 3b).

**Fig. 4:**
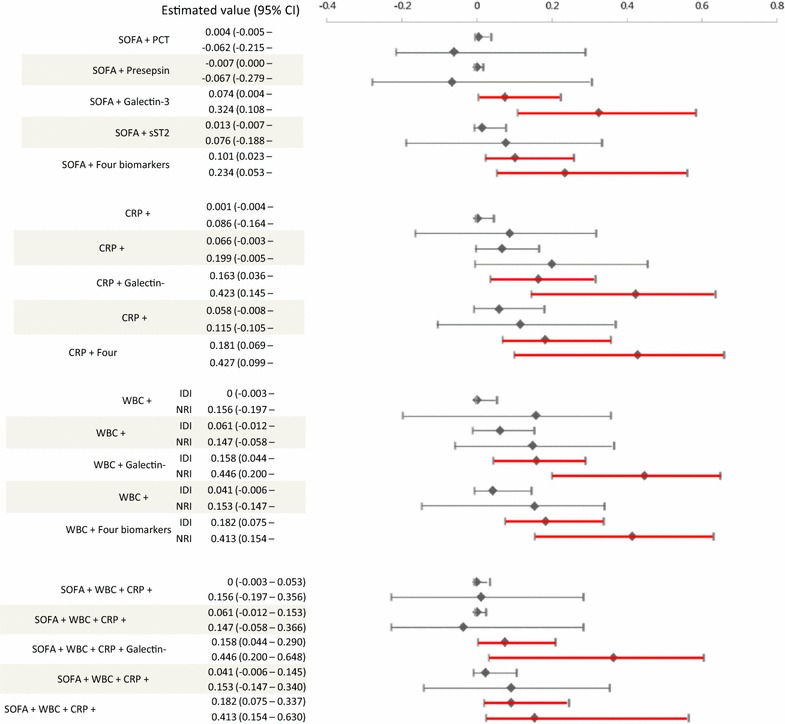
Multimarker approach to predict 30-day mortality in sepsis. Reclassification analyses of biomarkers and SOFA score using NRI and IDI. The rhombi mean estimated values and lines mean 95% CI. Abbreviations: *PCT* procalcitonin; sST2, soluble suppression of tumorigenicity 2, *ROC* receiver operating characteristics, *SOFA* sequential organ failure assessment, *IDI* integrated discrimination improvement, *NRI* net reclassification improvement, *CRP* C-reactive protein, *WBC* white blood cells, *CI* confidence interval

In reclassification analyses, the four biomarkers added on top of SOFA score, CRP, and WBC showed increased prognostic values than SOFA score, CRP, and WBC alone. Among the four biomarkers, only galectin-3 showed added values on top of SOFA score, CRP, and WBC (Fig. 4).

## Discussion

This study evaluated the prognostic utility of PCT, presepsin, galectin-3, and sST2, and their combinations in septic patients. As new sepsis definition became available in early 2016, our study population, who were recruited according to the Surviving Sepsis Campaign 2012, were reclassified as sepsis and septic shock; the patients who could not fulfill the new criteria were excluded [1, 16, 17].

In the present study, new biomarkers of presepsin, galectin-3, and sST2 were better than PCT for the prediction of 30-day mortality, and differently from PCT, they were higher in non-survivors than in survivors. SOFA score also showed such a difference. Of note, galectin-3 was the strongest risk predictor of 30-day mortality.

PCT is the US FDA-approved biomarker for risk assessment of septic patients, and its increase over time can be an aid in assessing the cumulative 28-day risk of all-cause mortality for patients diagnosed with septic shock [4, 5]. Presepsin has shown good diagnostic performance in predicting bacteremia and bacterial DNAemia in patients with suspected sepsis, and both PCT and presepsin have shown similar performances for predicting 28-day mortality [7, 23, 24]. In our data, PCT was the only marker that showed no concentration difference between the survivors and non-survivors and no predictive power for 30-day mortality. These findings suggest that PCT is less prognostic than the other three biomarkers and SOFA score.

According to the Sepsis-3 definition, at least two independent progressive organ dysfunctions are required for the diagnosis of sepsis, and septic shock is a subset of sepsis with underlying circulatory and cellular abnormalities. Both galectin-3 and sST2 were introduced as cardiac biomarkers, and they could predict worsened outcome of HF [10–12, 25, 26]. In addition to HF, galectin-3 and sST2 are independent biomarkers of inflammation, fibrosis, and cardiac stress. They are not specific for a distinct medical condition but rather represent general marker of mortality [13, 15, 27]. Our findings are in line with the previous findings and new sepsis definition, and galectin-3 and sST2 may have reflected circulatory abnormalities in this study population.

The present study addressed that multi-marker approach may be an aid for the prognosis prediction in septic patients. Our results are novel with respect to combined use of PCT, presepsin, galectin-3, and sST2 as markers of sepsis per se and organ dysfunction. We also combined these biomarkers with clinical variables, representatively SOFA score. As the number of above cutoff values increased from 0 to 5, the 30-day mortality increased in a stepwise pattern. Of note, group 0 had no event of mortality, and group 5 showed higher HR compared with groups 1, 2, and 3.

SOFA score was suggested 20 years ago to describe multiple organ failures in sepsis, using six different subscores (ranging from 0 to 4) for each organ. In general, there was an increasing mortality rate with a greater SOFA score and a good distribution of patient numbers among the different scores. However, SOFA scoring system had acknowledged limitations in terms of variables used and mortality discrimination, especially for cardiovascular and coagulation systems [28]. Accordingly, there is a room for further improvement of this scoring system with incorporation of promising biomarkers. Our data showed a significantly added value of promising biomarkers on top of SOFA score as well as established biomarkers of CRP and WBC. Of note, galectin-3 showed the strongest prognostic value added on top of clinical variables and established biomarkers. Several recent studies also explored diagnostic or prognostic usefulness of combined biomarkers in heterogeneous, critically ill settings [29–31].

This study has several limitations. We focused on the comparison of PCT, presepsin, galectin-3, and sST2 concentrations with 30-day mortality; so, we did not investigate the distribution of these biomarkers in relation to the specific bacteriological identification or antibiotic consumption. In addition, we did not perform follow-up measurements of these biomarkers. Due to the limited volume of blood samples, we could not measure other biomarkers, including natriuretic peptides, high-sensitive cardiac troponins, interleukin-6, which are known to be strong prognosticators in septic patients. We used SOFA score only; we did not use other clinical variables, such as Simplified Acute Physiology Score **(**SAPS) II, SAPS III, and/or Acute Physiology and Chronic Health Evaluation II scores. Further studies are encouraged to elucidate the clinical usefulness of the combination of these biomarkers.

## Conclusion

In conclusion, this study explored the prognostic utility of PCT, presepsin, sST2, and galectin-3 in septic patients. Compared with PCT, the other novel biomarkers showed superior prognostic performances, and their combined use reflected clinical outcome. Multi-marker approach using PCT, presepsin, sST2, and galectin-3 seems to be objective and useful for the prognosis prediction in septic patients.

## Abbreviations

AUC: area under the curve
CI: confidence interval
CLSI: Clinical and Laboratory Standards Institute
CRP: C-reactive protein
Cr: creatinine
ED: emergency department
FDA: Food and Drug Administration
GFR: glomerular filtration rate
HF: heart failure
HR: hazard ratio
IDI: integrated discrimination improvement
ICU: intensive care unit
IQR: interquartile range
NRI: net reclassification improvement
PCT: procalcitonin
RR: relative risk
ROC: receiver operating characteristic
SOFA: Sequential (Sepsis-related) Organ Failure Assessment
SAPS: Simplified Acute Physiology Score
sST2: soluble suppression of tumorigenicity 2
WBC: white blood cells
